# Hypothesis and Theory: A Two-Process Model of Torpor-Arousal Regulation in Hibernators

**DOI:** 10.3389/fphys.2022.901270

**Published:** 2022-06-20

**Authors:** Thomas Ruf, Sylvain Giroud, Fritz Geiser

**Affiliations:** ^1^ Research Institute of Wildlife Ecology, Department of Interdisciplinary Life Sciences, University of Veterinary Medicine, Vienna, Austria; ^2^ Centre for Behavioural and Physiological Ecology, Zoology, University of New England, Armidale, NSW, Australia

**Keywords:** cycles, interbout euthermia, metabolic rate, hourglass mechanism, periodic arousal, circadian rhythms

## Abstract

Hibernating mammals drastically lower their metabolic rate (MR) and body temperature (T_b_) for up to several weeks, but regularly rewarm and stay euthermic for brief periods. It has been hypothesized that the necessity for rewarming is due to the accumulation or depletion of metabolites, or the accrual of cellular damage that can be eliminated only in the euthermic state. Recent evidence for significant inverse relationships between the duration of torpor bouts (TBD) and MR in torpor strongly supports this hypothesis. We developed a new mathematical model that simulates hibernation patterns. The model involves an hourglass process H (Hibernation) representing the depletion/accumulation of a crucial enzyme/metabolite, and a threshold process H_thr_. Arousal, modelled as a logistic process, is initiated once the exponentially declining process H reaches H_thr_. We show that this model can predict several phenomena observed in hibernating mammals, namely the linear relationship between TMR and TBD, effects of ambient temperature on TBD, the modulation of torpor depth and duration within the hibernation season, (if process H_thr_ undergoes seasonal changes). The model does not need but allows for circadian cycles in the threshold T, which lead to arousals occurring predominantly at certain circadian phases, another phenomenon that has been observed in certain hibernators. It does not however, require circadian rhythms in T_b_ or MR during torpor. We argue that a two-process regulation of torpor-arousal cycles has several adaptive advantages, such as an easy adjustment of TBD to environmental conditions as well as to energy reserves and, for species that continue to forage, entrainment to the light-dark cycle.

## Introduction

During hibernation, mammals reduce their metabolic rate (MR) often down to ≤5% of basal metabolic rate (BMR) (Ruf and Geiser, 2015). In most species this decrease in MR is accompanied by a reduction of body temperature (T_b_) to values just above ambient temperature (T_a_). During hibernation, T_b_ decreases with ambient temperature (T_a_) over a wide range, but a species specific setpoint T_b_ is defended by increasing heat production and energy expenditure once T_a_ falls below the setpoint T_b_ (Wyss, 1932; Florant and Heller, 1977; Geiser et al., 1990; Buck and Barnes, 2000; Munro and Thomas, 2004; Tøien et al., 2011; Richter et al., 2015; Ruf and Geiser, 2015; Geiser, 2021).

**FIGURE 1:**
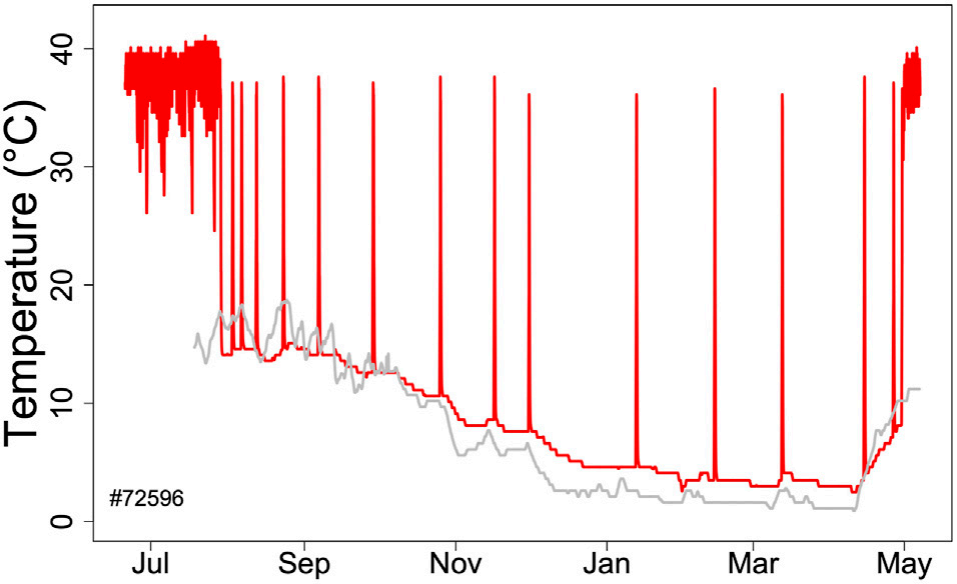
Body temperature record (red line) during hibernation in an edible dormouse (*Glis*) free-living in the Vienna Woods (Austria). Core body temperature was recorded using a temperature logger (DS 1922L, Maxim, Dallas, United States; resolution 0.5°C) at ∼ hourly intervals. The gray line shows soil temperature at the approximate depth (60 cm) of dormice hibernacula.

**FIGURE 2:**
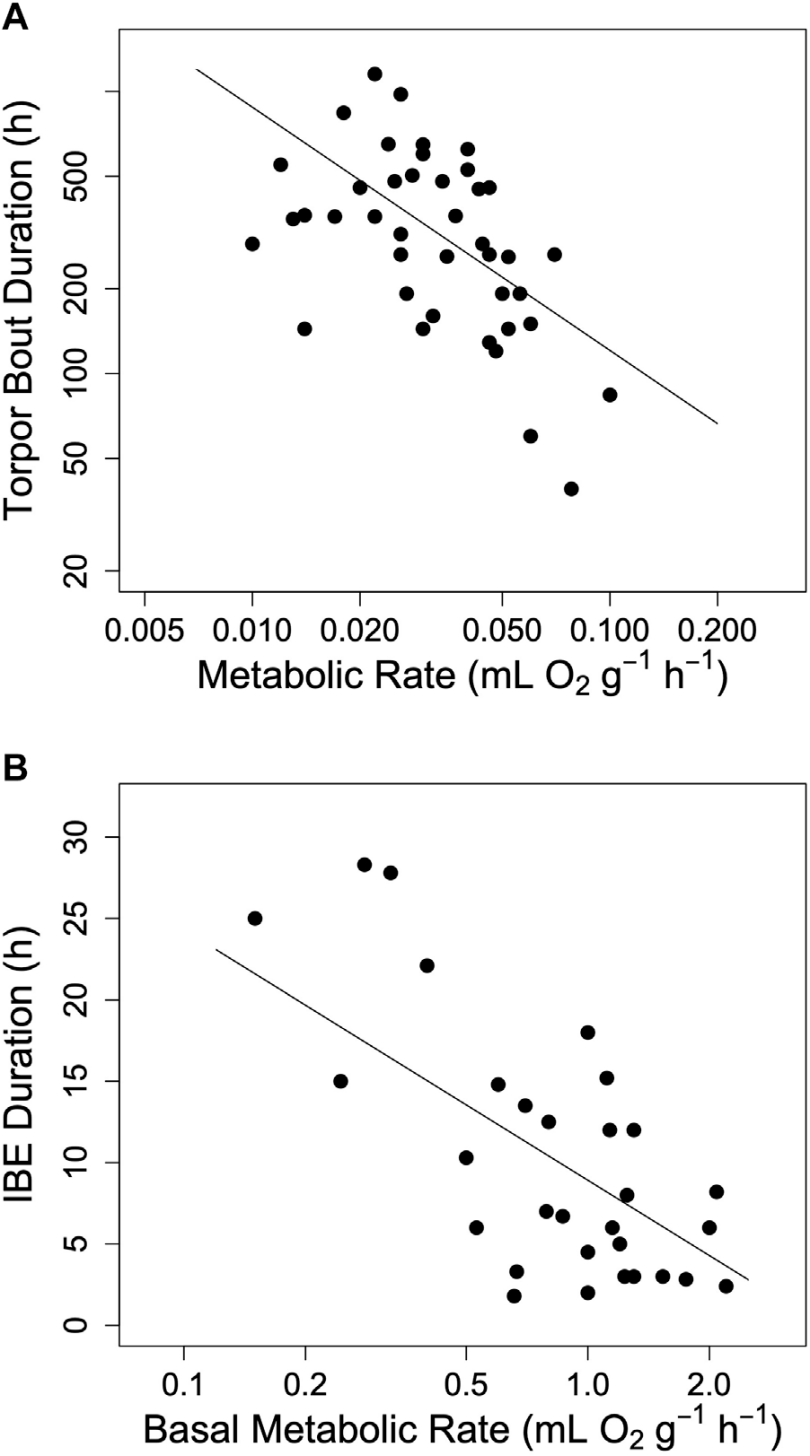
The impact of metabolic rate on hibernation in mammals. **(A)** The maximum duration of torpor bouts (TBD) among hibernating mammals as a function of minimum metabolic rate in torpor (log10 TBD = 1.22–0.862 log10 TMR, t = −4.56, *p* < 0.0001, *r* ^2^ = 0.20). **(B)** The duration of interbout euthermia (IBE) as a function of basal metabolic rate (IBE = 8.92–15.39 log10 BMR, t = −3.80, *p* < 0.001, *r* ^2^ = 0.50). Regression lines in both panels a and b were computed using phylogenetically informed regression procedures. Data were obtained from Table 1 in Ruf and Geiser (2015).

Hibernators enter a state of torpor that can be maintained for several weeks. However, they typically do not stay at low MR and T_b_ throughout winter. Apart from a few species that can continually hibernate at T_b_s of approximately 20–30°C (Dausmann et al., 2004; Tøien et al., 2011; Lovegrove et al., 2014; Ruf and Geiser, 2015) hibernating mammals regularly rewarm from to the euthermic state during so-called spontaneous arousals (Ar; Figure 1). The maximum duration of torpor bouts (TBD) is species-specific and varies from ∼3 to 98 days (Figure 2; Ruf and Geiser, 2015). Arousals and subsequent intervals of interbout euthermia (IBE) are responsible for at least 70% of the total energy expenditure over winter (Wang, 1979).

However, since the first discovery of these spontaneous “periodic changes” of T_b,_ their function has remained unclear (Hall, 1832). Among other hypotheses, it has been suggested that hibernators rewarm in order to sleep (Daan et al., 1991; Trachsel et al., 1991), but this explanation was later questioned (Larkin and Heller, 1998; Strijkstra and Daan, 1998). It was also suggested that animals rewarm to combat pathogens (Prendergast et al., 2002), or to restore enzymes required for cardiac function at low T_b_ (Ruf and Arnold, 2008; Giroud et al., 2013). Also, the clock mechanisms that control the timing of torpor and arousal within the hibernation season are entirely unknown, and even their fundamental nature is a question of debate (Malan, 2010; Ruf and Geiser, 2015).

For a long time, it had been assumed that the torpor-arousal cycle is driven by so-called hourglass mechanism. This hypothesis assumes the development of a metabolic imbalance during torpor, for instance, the depletion of a crucial metabolite during torpor that can be produced only in the euthermic state (Fisher, 1964; Twente and Twente, 1968; Galster and Morrison, 1976; Lyman, 1982; French, 1985; van Breukelen and Martin, 2002; Carey et al., 2003; Martin and Epperson, 2008; Strijkstra et al., 2008). However, if an houglass mechism is indeed at work, it seems that at low T_b_ the continued degradation and depletion of metabolites is much more likely than their energy-consuming production and accumulation.

The hourglass hypothesis seemed to be supported by the observation that an increase in MR during torpor, due to animals defending a setpoint T_b_ at very low T_a_, is associated with a shortening of TBD (Geiser and Kenagy, 1987; Geiser and Kenagy, 1988; Buck and Barnes, 2000). The relationship between MR and TBD was also supported by the finding that both TBD and the duration of IBE among mammals indeed decrease as MR increases (Ruf and Geiser, 2015; see also Figure 2). Finally, we found that within a species, the garden dormouse, the duration of torpor bouts was highly dependent on MR (Ruf et al., 2021; Supplementary Figure S1). This effect of torpor oxygen consumption on TBD strongly indicated an hourglass mechanism of torpor control.

However, Malan (2010) argued that the absence of an effect of body mass on TBD is reason to refute the hourglass hypothesis. Indeed, as MR is usually strongly affected by body mass, it seems logical to assume that independence of TBD from body mass also should reflect independence of the torpor-arousal cycle from MR. Instead of an hourglass mechanism, Malan, (2010) therefore proposed the existence of a specialized circadian clock that governs torpor-arousal cycles. However, subsequent, comprehensive comparisons showed only an extremely weak dependency of torpor metabolic rate on body mass among hibernating mammals (Ruf and Geiser, 2015). Also, if each torpor episode represented a single circadian cycle (Malan, 2010; van Breukelen and Martin, 2015), this would require a non-temperature compensated clock, which contradicts the empirical evidence (Rawson, 1960; Zimmerman et al., 1968). Moreover, in free-living dormice, for example, a single circadian day would have to be lengthened from 24 h to >800 h during midwinter (Hoelzl et al., 2015). Thus, considering the changes required, this scenario assuming a governing role of the circadian system is rather unlikely.

A pure hourglass mechanism seems insufficient, however, to explain a variety of observations that point to an involvement of the circadian system in the temporal control of torpor-arousal cycles. A number of studies have reported circadian patterns in the timing of torpor bouts or arousal intervals especially in species that perceive daylight throughout winter (Daan, 1973; French, 1977; Pohl, 1987; Twente and Twente, 1987; Canguilhem et al., 1994; Grahn et al., 1994; Wollnik and Schmidt, 1995; Pohl, 1996; Waßmer and Wollnik, 1997; Körtner et al., 1998; Turbill et al., 2008). For instance, under conditions of a light dark cycle (LD), histograms of TBDs or the intervals between subsequent arousals in garden dormice and pocket mice show discrete peaks at multiples of 24 h (Supplementary Figure S3). It is easy to envision how the circadian components in the timing of torpor could result from the interaction of an hourglass mechanism with a circadian oscillation. Qualitative models illustrating this idea were already suggested by French, (1977) and Geiser et al. (1990). Here, we follow up on these hypotheses by developing a mathematical model based on two processes, an hourglass mechanism that determines the principal periodicity of torpor-arousal cycles, and a threshold process that undergoes both seasonal and circadian oscillations. Obviously, this model was inspired by the two-process model of sleep regulation (Borbély, 1982; Daan et al., 1984). We show that our model can predict a number of real-world observations, including the dependency of TBD on mean MR, so called test-drops around hibernation onset, the absence of a circadian rhythm in MR and T_b_ during hibernation, and circadian patterns in the timing of torpor bouts under certain conditions.

## The Model

**FIGURE 3:**
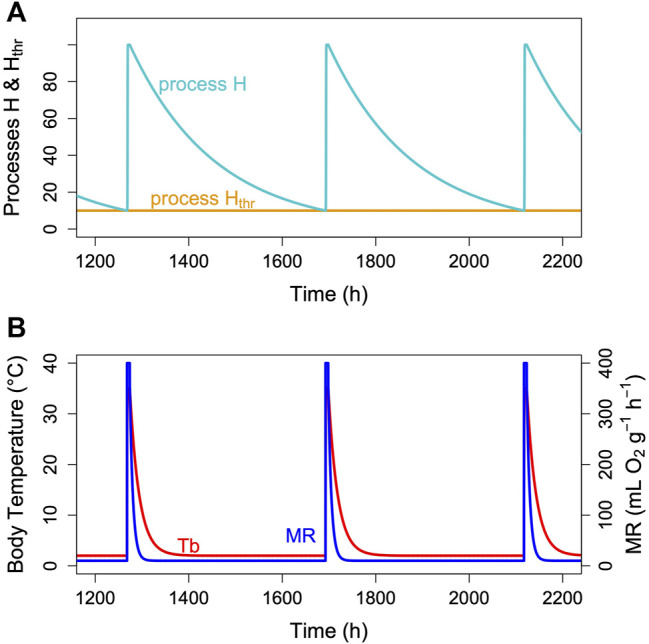
The two-process model of torpor-arousal regulation. **(A)** Process H (turquoise line) alternates between a depletion phase and a recovery phase. The time constant k (Eq. 1) is kept at 0.005. Recovery during arousal is initiated once process H reaches the threshold H_thr_ (orange line). In this most basic model, process H_thr_ is just a constant threshold. **(B)** Resulting simulated T_b_ and MR pattern in an average hibernator.

The hibernator’s MR and its T_b_ are measured variables, which change over time. MR is usually determined from oxygen consumption and CO_2_ production that are converted to MR (Lighton, 2008). T_b_ is typically measured using thermistors, transmitters or thermo-loggers. For the purpose of a model, it is sufficient to predict the time course of MR, particularly TBD, which lasts from the decrease of MR at torpor entrance to the end of its decline at which arousal is initiated (Figure 3A). Hence, TBD is the main variable predicted. Its pendant in reality is the measured time from torpor entrance to the next arousal (Figure 3B). As hibernation is often described in terms of T_b_ only, we modelled its time course for illustration purposes. Both in reality and in the model, T_b_ follows MR in a damped manner, largely depending on the size and insulation of the animal (e.g., Ortmann and Heldmaier, 2000; Giroud et al., 2018; Geiser, 2021).

Hibernation is characterized by an alteration between metabolic reduction that may last several hundred hours and rapid, only up to 44 h lasting arousals (Ruf and Geiser, 2015). We assumed that this alteration is an hourglass process based on the loss of a crucial enzyme or metabolite over time, which is followed by a rapid restoration during arousal (Figure 3). However, the model cannot really distinguish this from a potential accumulation of a toxic metabolite. To model the depletion phase during an hourglass process (H) we used a simple exponential function of the form:

$$H=\mathrm{H0}\text{ ∗}e^{-\mathrm{k* t}}$$ (1)

Where ***H*** is the value of process H at time t (note that we use bold italic faces to indicate model variables). ***H0*** is the starting value of ***H*** at the beginning of torpor entrance (always 100%). The parameter ***k*** is a time constant determining the speed of the decline, and t is the time (in h) since torpor onset. In the model runs described below we varied k as stated in the figure legends leading to TBDs between ∼1 day to several weeks, depending on the question. Parameter ***k*** was chosen so as to result in realistic TBDs (see Figure 2). As ***H0*** is always 100% at torpor onset, ***k*** is the only parameter in Eq. 1. The model behavior is fully controlled by H. The time constant ***k*** is proportional to the mean metabolism in torpor, in fact, it is determined by that fraction of TMR that is linked to process H. We argue (see below) that at T_a_ ≤ 0°C an increasing part of TMR is used for heat generation, which leaves only a smaller portion for process H. TBD is simply determined from the duration of a decrease in ***H*** (see Supplementary Material). Over several series of torpor bouts, there is a linear relationship between mass specific TMR and TBD (Figure 6). The purpose of the fitting process (Supplementary Material) is to identify an intercept and a slope that minimize the squared deviation from real and predicted torpor durations.

The main purpose of the shape of function H during entry and arousal was simulation of a slow decline of a crucial metabolite and its sharp increase during warm-up. We would argue that H actually represents an exponential process because in the short term the decrease is partly due to the effect of temperature as described by the Arrhenius equation. In the long term we simulate the decline (and no production at low T_b_) of a population of metabolically active units, which is a typical exponential function.

Unless the nature of the critical component is identified and measured, we do not know the exact relationship between the time courses of TMR and process H within a single torpor bout. However, we conclude (Figure 2, Ruf et al., 2021) that the higher TMR at a given mass, the higher k, the faster is the decline of H and vice versa. Also, the critical component during hibernation is still declining (process H) while TMR is already roughly constant. Thus, TMR changes much more quickly than H. TMR and is also reduced faster than T_b_ and changes in T_b_ only result from the time course of MR. We give an example of the approximate time course of process H, MR and T_b_ for an average hibernator in Figure 3. For a real-world example see Figure 1 in Ruf et al. (2021).

As soon as ***H*** reached a threshold value (***H***_***thr***_), we terminated the entrance loop, assigned ***H0*** the current value of ***H*** and used a logistic function to simulate the arousal phase:

$$H=\frac{100-\mathrm{HO}}{1+e^{10+k_{\mathrm{up}}\mathrm{* t}}}+\mathrm{H0}$$ (2)

Here, ***k***_***up***_ is the time constant for the increase of ***H***, and ***t*** is the time in hours since arousal onset. The warmup was always modelled much faster than the declining phase (k_up_ = 8).The arousal and IBE loop was terminated once ***H*** reached or exceeded 100%, and the next torpor entrance phase was started. Thus, once H started to increase it only stopped after reaching 100%. The purpose of the shape of function ***H*** during arousal was simulation of the rapid restoring of a crucial substance at high temperature. Among other functions, it is used to describe the assembly of objects.

Process ***H*** simulates depletion, for instance, that is faster when ***k*** is large (and TMR is high). The threshold ***H***_***thr***_ marks a value that, once it is reached, triggers a complete arousal. It causes process ***H*** to change its direction until it is completed again (100% are reached) simulating, for instance, the rebuilding of a crucial molecule. ***H***_***thr***_ is actually a certain value of H on the same scale. The threshold ***H***_***thr***_ in the simplest case can be constant, or it may be changing seasonally and/or daily.

To model T_b_ patterns that mimic actual time courses we used the same functions but different constants. Note that these functions are only used for illustrative purposes and are not part of the actual model to predict TBD. For torpor entrance, T_b_ was simulated using

$$\mathrm{Tb}=\left(\mathrm{Tb0}-Tb_{\min}\right)\text{∗}e^{-k_{t}*t}+Tb_{\min}$$ (3)

Where ***Tb*** is the value of ***Tb*** at time ***t***, ***Tb0*** is the starting ***Tb*** at torpor onset (set to 35°C), ***kt*** is the temperature time constant, and ***Tb***_***min***_ is the minimum starting temperature. Note that we use the notation ***Tb*** when referring to the model variable, but use T_b_ when referring to actual body temperature of animals. To simulate T_b_ changes during arousal we first assigned the last entrance value of ***Tb*** to ***Tb0***, and subsequently computed rewarming ***Tb*** s using

$$\mathrm{Tb}=\frac{35-\mathrm{TbO}}{1+e^{1+kt_{\mathrm{up}}\mathrm{* t}}}+\mathrm{Tb0}$$ (4)

Note that ***t***, again, means the time since arousal onset, as determined by process ***H*** reaching the threshold ***H***_***thr***_. This rapid increase of ***Tb*** and its reaching plateau resembles actual records of T_b_ in hibernators during arousals (Figure 1, Ortmann and Heldmaier, 2000; Geiser, 2021; Ruf et al., 2021). To match the time course of realistic T_b_ changes the parameter ***kt***_***up***_ was always kept at the default value of 2.0 starting with an initial T_b_ (***Tb***_***min***_) of 2°C.

***Tb*** can also be used to determine the approximate ***TBD*** from the time interval of ***Tb*** dropping below 30°C and returning to above 30°C. Similarly, arousal-to-arousal (Ar-Ar) intervals were computed from the time between subsequent transitions of ***Tb*** < 30°C to ***Tb*** > 30°C. For more details, see Supplementary Material.

**FIGURE 4:**
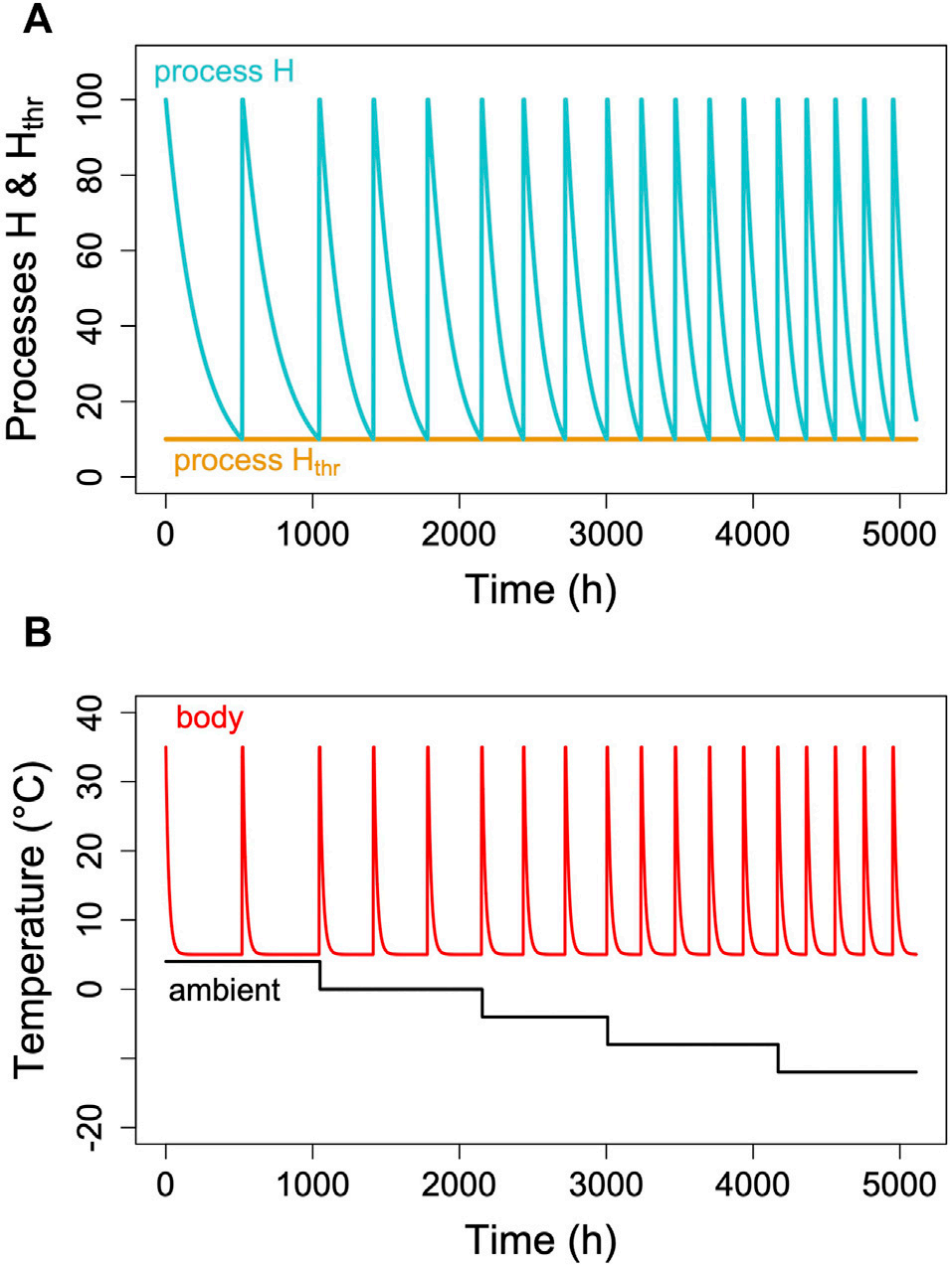
Model of the effects of T_a_ decreasing below T_b-min_. Decreasing T_a_ is accompanied by increased thermoregulatory heat production and shortening of TBD. **(A)** The threshold is constant while the time constant of the depletion phase of process H increases with decreasing T_a_. In the example the time constant k for each bout is increased from 0.0044 to 0.014 as T_a_ dropped. **(B)** The resulting pattern in T_b_ as T_a_ declines.

**FIGURE 5:**
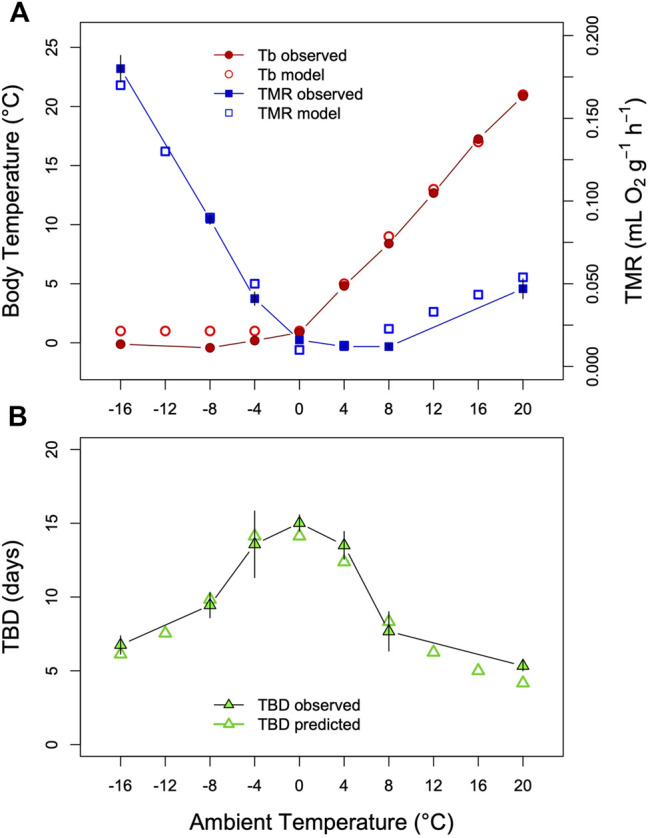
Model of TBD regulation ground squirrels. **(A)** T_b_ and TMR measured in Arctic ground squirrels hibernating at T_a_s between −16°C and +20°C (filled symbols) as well as model variables (open symbols). Data were obtained from Table 2 and Figure 2 in Buck and Barnes (2000). **(B)** Comparison of TBD as predicted from the model (open triangles) with TBD determined in a separate group of arctic ground squirrels (filled triangles). To model a partial effect of TMR on TBD as T_b_-T_a_ gradients increase (see text) the fraction of TMR determining TBD was estimated at 20%, parameter ***k*** was estimated as ***k*** = 0.004 + TMR*0.485; TMR = TMR*0.20 for *Ta*<=+4°C). This resulted in an approximately inversely linear relationship between TBD and the TMR-transformation. To model TBD over the entire range, *Tb* was simulated by setting it to *Ta*+1 above *Ta* = 0°C, and keeping it constant at lower *Ta*s. TMR was simulated by a slight exponential increase with *Tb* at *Ta* > 0°C, and a linear increase with *Tb-Ta* at lower *Ta*s (both fitted to the data in Figure 5A).

**FIGURE 6:**
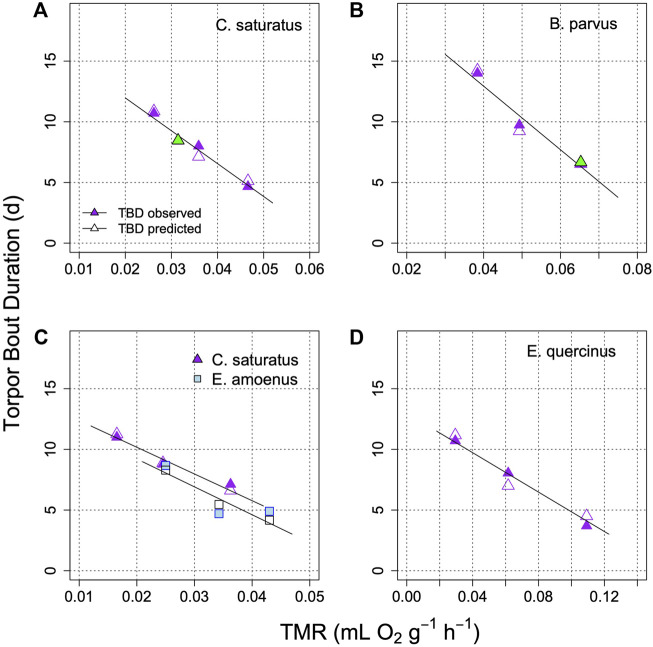
Model of TBD regulation in golden mantled ground squirrels **(A)**; Geiser and Kenagy, 1988), mountain pygmy possums **(B)**; Geiser and Broome, 1993), squirrels and chipmunks **(C)**; Geiser et al., 1990), and garden dormice **(D)**; Ruf et al., 2021). Each graph shows TBD as a function of TMR for different parts of the hibernation season, or at different T_a_ (means). Open symbols are model predictions of TBD. In two cases (green symbols in A and B) the fraction of TMR determining TBD was estimated for minimizing (TBD_measured-_TBD_predicted_^2^). This proportion estimate was 18% in **(A)** and 24% in **(B)**. TBD was computed to an artificial ***H***_***thr***_ of 10%. The parameter ***k*** was −0.025 + 0.44*TMR in A; −0.045 + 0.295*TMR in B; 0.05 + 0.235*TMR in C for *S. saturus*; −0.0045 + 0.645*TMR in C for *E. amoenus* 0.004 + 0.16*TMR in **(D)**.

To model the long-term relationship between MR during torpor bouts and TBD and for comparison with published results, we digitized graphical representations of results in Daan, (1973); Figure 10 Buck and Barnes, (2000); Figures 1, 2, Geiser et al. (1990); Figures 3, 4, Geiser and Broome, (1993); Figure 2, Geiser and Kenagy, (1988); Figure 2, and used own data Ruf et al. (2021). Data were converted from published graphs to numbers using package “digitize” (Poisot, 2011). Bout duration was modeled from TBD = p1+TMR*p2. In cases when TMR under cold load (T_a_ ≤ 0°C) was clearly elevated due to thermoregulation, we additionally determined a parameter p1 giving an estimate of the fraction of TMR contributing to TBD (Figures 5, 6). Parameters were always estimated by minimising (TBD_observed_ - TBD_predicted_)^2^, see Supplementary Material.

Phylogenetically informed regressions were computed using generalized least squares and functions in package “ape” (Paradis et al., 2004). All computations were carried out in R 4.1.2 (R Core Team, 2021).

## Metabolism and the Torpor Arousal Cycle

A basic tenet of the model proposed here is that short TBD is associated with high minimum TMR. There can be little doubt that this is the case among hibernating species (Figure 2). However, average MR during torpor may also vary within species. For instance, when T_a_ falls well below the setpoint T_b_ (Florant and Heller, 1977; Munro and Thomas, 2004) hibernators increase thermoregulatory heat production in a tightly controlled manner (for instance, Wyss, 1932; Florant and Heller, 1977; Geiser et al., 1990). Large T_b_-T_a_ gradients and hence increased TMRs are accompanied by a shortening of TBD (for instance, Geiser and Kenagy, 1988; Buck and Barnes, 2000). This can be modelled by simply making the time constant k of process H, which is the major determinant of TBD, a linear function of T_a_ (Figure 4).

In hibernators maintaining T_b_ above the setpoint there is also a strong decrease of TBD with increasing T_b_ (Twente and Twente, 1965; Geiser and Kenagy, 1988; Buck and Barnes, 2000; Ortmann and Heldmaier, 2000; Bieber and Ruf, 2009; Nowack et al., 2019). According to the hourglass hypothesis, this effect would be expected from Arrhenius effects at higher T_b_ or increased T_b_-T_a_ gradients leading to elevated MR. However, as has been pointed out earlier (Geiser and Kenagy, 1988; Buck and Barnes, 2000; Malan, 2010), the relationship between TMR and TBD seems more complicated than suggested by Figure 2 and Supplementary Figure S1. In a study by Buck and Barnes, (2000) on arctic ground squirrels, for instance, at T_a_s above T_b_-min (+4°C) TBD was continually shortened while TMR showed only a much smaller increase up to T_a_ +20°C compared with the rise of TMR below T_a_ 4°C (Figure 5). It should be noted, however, that TMR and TBD in these experiments were determined in separate groups of arctic ground squirrels (Buck and Barnes, 2000). Nevertheless, the same asymmetry in the relationship between TBD an MR before and after the onset of thermoregulatory heat production was also found in hibernating golden-mantled ground squirrels (Geiser and Kenagy, 1988). This may mean that T_b_ as such, in addition to MR, contributes to determining TBD (Geiser and Kenagy, 1988; Buck and Barnes, 2000). In this context, it has been hypothesized that the crucial variable determining TBD could be brain temperature, which might be higher than core T_b_ and increasing during thermogenesis at increasing T_b_-T_a_ gradients (Buck and Barnes, 2000). During hibernation there can be considerable gradients of T_b_, for instance between brain and abdomen (Barnes, 1989; Buck and Barnes, 2000). However, without continuous records of brain temperature and TBD in the same animals this hypothesis is impossible to test. Moreover, brain temperature alone does not explain the significant role of torpor MR on TBD (Figure 2, Supplementary Figure S1).

An alternative explanation for these results is that overall TMR may be only a proxy for the actual rate of process H. During cold exposure (at T_a_ < setpoint-T_b_) hibernators increase heat production predominantly in specialized tissues, such as brown adipose tissue and skeletal muscle (Nowack et al., 2017), which conceivably may not be the main tissues of an accumulating metabolic imbalance. Thus, the fraction of metabolism allocated to pure heat production, in contrast to ‘basal’ TMR without thermogenesis, may not contribute equally to the formation of an imbalance during torpor.

According to this view, the increase in TMR that accelerates the metabolic imbalance is only a fraction of total TMR. This can be incorporated in the model by, for instance, limiting the effect of TMR on the time constant of process H to a certain proportion as heat production is increased at low T_a_ in hibernation. The resulting model-prediction of TBD fits the data of Buck and Barnes, (2000) quite well (Figure 5). This complication of the model may be unelegant but the results match the asymmetry of reality. After all, this asymmetry only reflects that we measure O_2_ consumption as the sum of all tissue-specific metabolic rates.

Applications of the model on the prediction of TBD from TMR in several further species, under controlled laboratory conditions is illustrated in Figure 6. This graph shows that the relationship between TBD and mean metabolism is indeed linear. It remains linear in the model when TMR determines the rate at which process H approaches the threshold. Interestingly, in all cases when the fraction of TMR devoted to process H was determined from the best fit, it was similar at 18%–24% (Figures 5, 6). It should be also noted that Figure 6 shows results from both placental mammals and a marsupial (B), hence the model may be generally applicable to mammals.

Another way by which T_a_ could influence TBD is *via* effects on the threshold process ***H***_***thr***_, rather than on the time constant of process H. This possibility has been suggested by Strijkstra et al. (2008), based on experiments with ground squirrels in which T_a_ was altered during torpor bouts. While it would be feasible to incorporate this modification into our model, we feel that, given the large scatter in the data collected by Strijkstra et al. (2008), the factual basis for introducing such a complication of the model is currently not solid enough. Clearly, more experimental work is necessary to test the idea that the acute T_a_ at the initiation of arousal determines TBD irrespective of prior changes in T_a_ during the torpor bout, or that TBD is even determined at the beginning of a bout (Strijkstra et al., 2008; Geiser and Mzilikazi, 2011).

As to the nature of process H little is known. Hibernation is largely powered by lipids (Dark, 2005) but there is nothing to indicate that lipid as fuels immediately determine TBD. It has been suggested that torpor duration is linked to glucose availability (Galster and Morrison, 1970) but this finding in a single species was later dismissed (Zimmerman, 1982). However, there is one further external factor that strongly affects TBD: altering the tissue concentrations of polyunsaturated fatty acids (PUFAs). In particular Linoleic acid (LA), through dietary uptake or endogenous remodeling of membranes can substantially affect TBD in hibernators (reviews in Munro and Thomas, 2004; Ruf and Arnold, 2008; Arnold et al., 2015). These effects are compatible with the hourglass hypothesis because it seems that substantial increases of TBD caused by increased incorporation of LA were always accompanied by decreases in TMR and/or minimal T_b_ (Geiser and Kenagy, 1987; Geiser and Kenagy, 1993; Geiser et al., 1994; Thorp et al., 1994). Hence, at least part of the effect of PUFAs on TBD appears to be mediated *via* lowering the T_b_ setpoint, and consequently TMR. This does not rule out however, that certain PUFAs may also directly affect the rate of process H during torpor, or its reversal during arousal.

In fact, it has been hypothesized that the slow degradation of the Ca^2+^-ATPase (SERCA) in cardiac myocytes, which is modulated by PUFA, may be the crucial factor that forces hibernators to rewarm periodically (Ruf and Arnold, 2008). This makes the depletion of this transmembrane protein a prime candidate for process H. SERCA is the key enzyme that ensures proper Ca^2+^ handling of myocytes and hence functioning of the heart, in particular, in deep hibernation. Non-hibernators exposed to severe hypothermia typically die from heart failure, that is, loss of contractibility of cardiac myocytes and ventricular fibrillation. Hence it has been suggested that hibernators can remain torpid until the need to restore SERCA activity *via* protein synthesis forces them to rewarm to euthermic temperatures (Ruf and Arnold, 2008).

Because high amounts of LA in the surrounding membrane increase the activity of this Ca^2+^-pump (Swanson et al., 1989; Giroud et al., 2013; Giroud et al., 2018), they should partly compensate for protein degradation over the torpor bout and increase the time a hibernator can remain torpid. Clearly, more research is needed to test this hypothesis, but if it turns out that there are indeed direct effects of PUFAs (or other substances) on the time constant of any accumulating metabolic imbalance, this outcome would not contradict an hourglass mechanism but merely indicate that factors in addition to MR can modulate its rate. However, since SERCA determines a vital function, heart rate, that is affected by experiments, it is difficult to test if SERCA indeed represents process H.

## Seasonal Modulation

**FIGURE 7:**
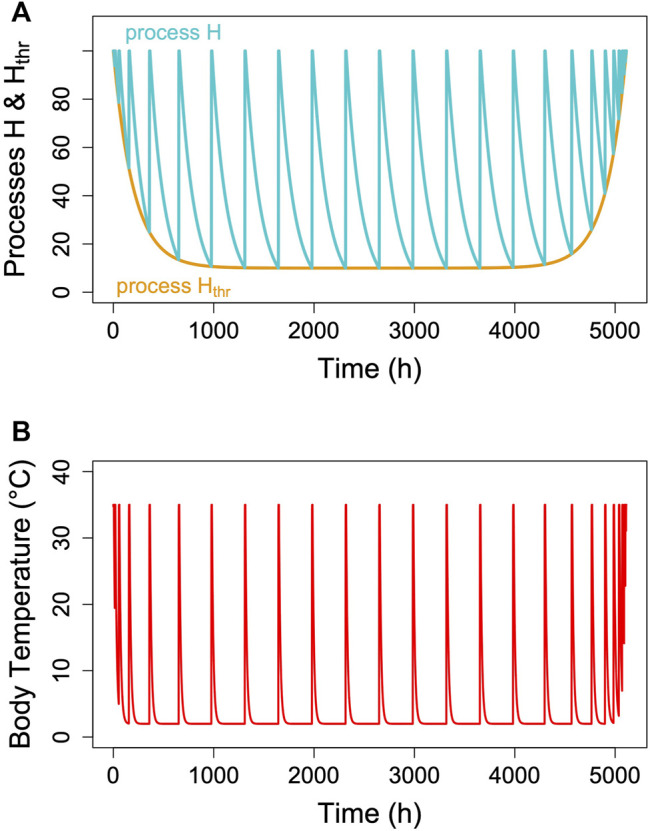
Seasonal modulation of process H_thr_ (orange line in panel **(A)**. The gradual lowering of the threshold H_thr_ after hibernation onset and gradual return to 100% towards hibernation end leads to simulated T_b_ patterns [panel **(B)**] with short, shallow torpor bouts during both of these phases. Parameter k was kept constant at 0.007 in the example.

Hibernators typically do not enter long, deep torpor bouts abruptly, but show gradually increasing depths and durations of torpor episodes (for instance, Figure 1). Often, the reverse pattern is observed at the end of the hibernation season (Figure 1). In free-living animals that experience continually changing T_a_, this can be partly explained by the effects of T_a_ and T_b_ on TBD (see discussion above). Gradual increases in depth and duration of torpor bouts early in the hibernation season, often called “testdrops”, also occur, however, under laboratory conditions at constant T_a_ (Strumwasser, 1959). The involvement of an endogenous component in the control of torpor is to be expected, since the entire cycle of seasonal alteration between activity and hibernation is governed by a circannual clock (Pengelley and Fisher, 1963; Pengelley and Asmundson, 1969). This seasonal component can be integrated into the model proposed here by introducing a seasonal modulation of the arousal threshold, process H_thr_ (Figure 7). This modification, which renders process H_thr_ one of the outputs of the circannual clock, immediately leads to the occurrence of “testdrops” (Figure 7). Given the model properties, increasing the threshold simultaneously affects both TBD and the minimum T_b_ reached, which reflects real world observations (Figure 1; Strumwasser, 1959).

Given its strong effect on TBD a prime candidate for slow, seasonal changes of hibernation patterns is membrane lipid composition. For example, there is remodeling of membranes in free-living alpine marmots (Arnold et al., 2011). This indicates selective trafficking of PUFA, probably governed by a circannual endogenous rhythm. There was an increasing rate of rewarming from torpor during winter that could be explained by the accumulation of PUFA composition. Edible dormice, on the other hand, when subjected to diets low in LA do not enter hibernation unless their membranes are remodeled in terms of PUFA composition (Giroud et al., 2018). This remodeling of membranes would, in terms of the current model, affect process H_thr_ and the sensitivity of process H towards arousal. Thus, they may well be the underlying reason for a seasonal modulation of arousal frequency.

## Does the Torpor Arousal Cycle Involve a Circadian Component?

**FIGURE 8:**
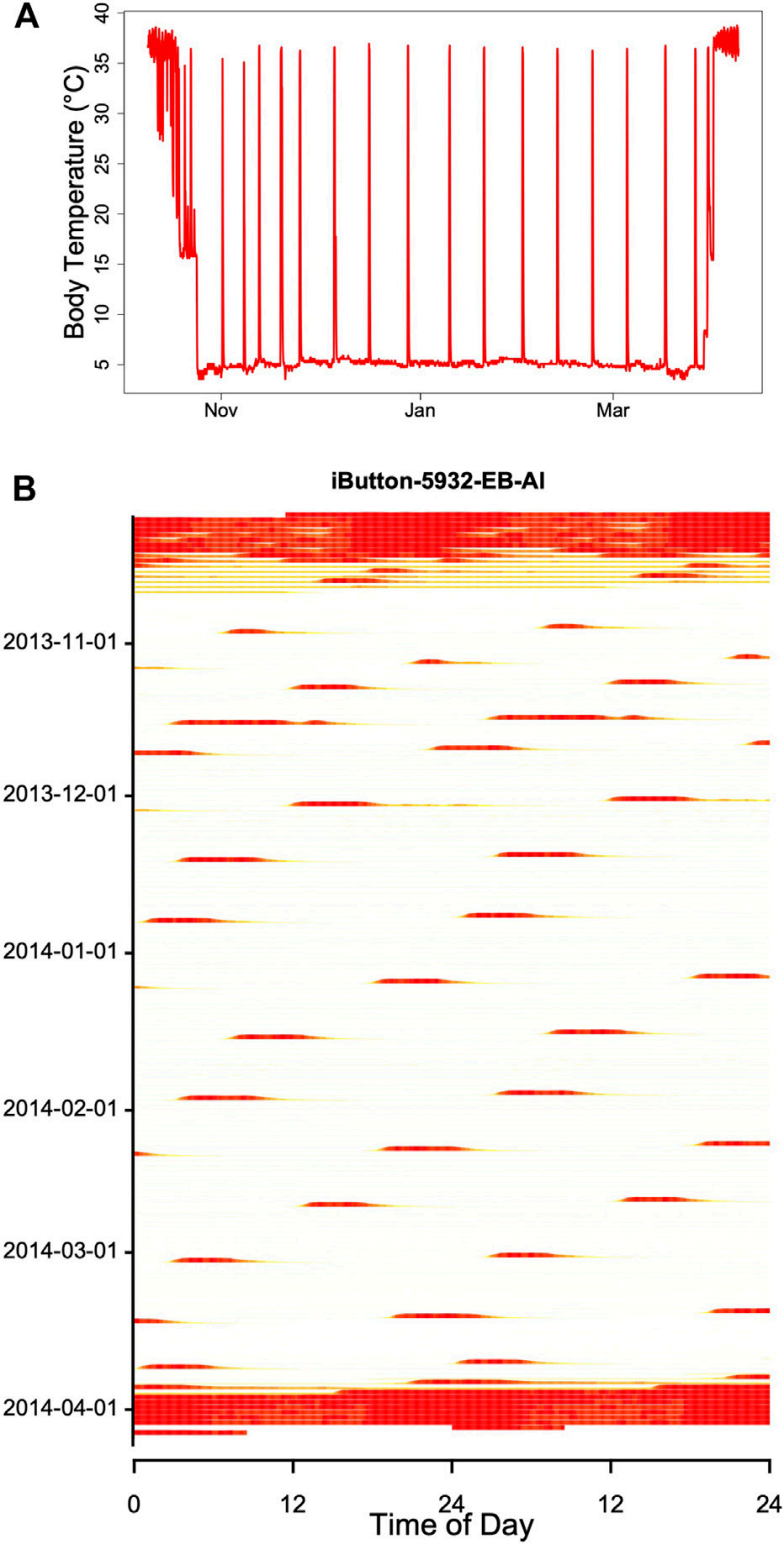
Record of T_b_ during winter in a garden dormouse [Eliomys quercinus; **(A)**] kept under laboratory conditions in constant darkness at T_a_ 5 ± 1°C. **(B)** The same data as in **(A)**, but double-plotted in an actogram-like style. On each horizontal line T_b_ is plotted over two consecutive days on a heat scale, with white corresponding to minimum T_b_ (∼5°C) and red corresponding to maximum T_b_ (∼39°C). During hibernation, the red bands originate from arousals, i.e., return to euthermia. These arousals show a free-running rhythm, that may suggest involvement of a circadian rhythm (but see Figure 9). Red bands at the beginning and end of hibernation indicate normothermia and short torpor bouts (see A).

**FIGURE 9:**
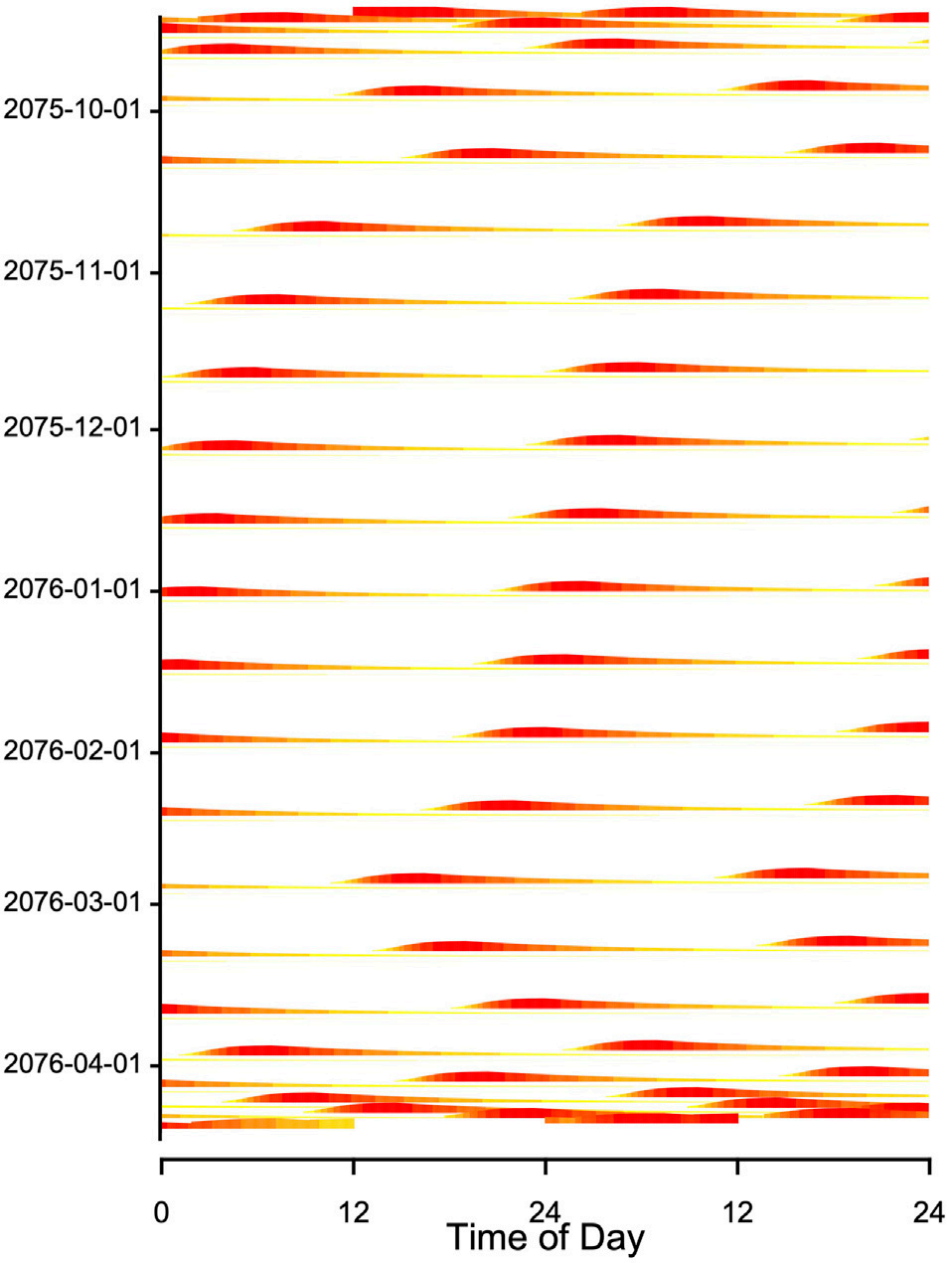
Double plot of simulated T_b_ from Figure 4. The model included a seasonally changing threshold H_thr_ and a fixed time constant (k = 0.007) of process H (plotted as in Figure 8B) During the main hibernation season, arousals (red horizontal bands) show a stable free-running rhythm, caused by a constant periodicity of process H. Note that the model generating these data contained no circadian component. Red and yellow bands at the beginning and end of the actogram indicate that the hibernation season starts and ends with shallow torpor bouts (see Figure 4).

When records of T_b_ or times of arousals during hibernation are plotted in an actogram–like fashion, they often show fairly regular patterns that resemble free-running circadian rhythms, albeit with long intermittent phases of low T_b_ (Figure 8; see also, for instance, Daan, 1973; French, 1977; Grahn et al., 1994; Hut et al., 2002). This may be suggestive of an ongoing, slowed-down circadian rhythm controlling the torpor-arousal cycle. However, it is easy to show that an hourglass mechanism, without the involvement of any circadian component, will result in such a pattern if T_a_ and hence TBD is fairly constant. For instance, Figure 9 shows an actogram-like representation of simulated ***Tb*** from a model in which process H alternates between 100% and a constant threshold T, without any variables containing circadian periodicity.

**FIGURE 10:**
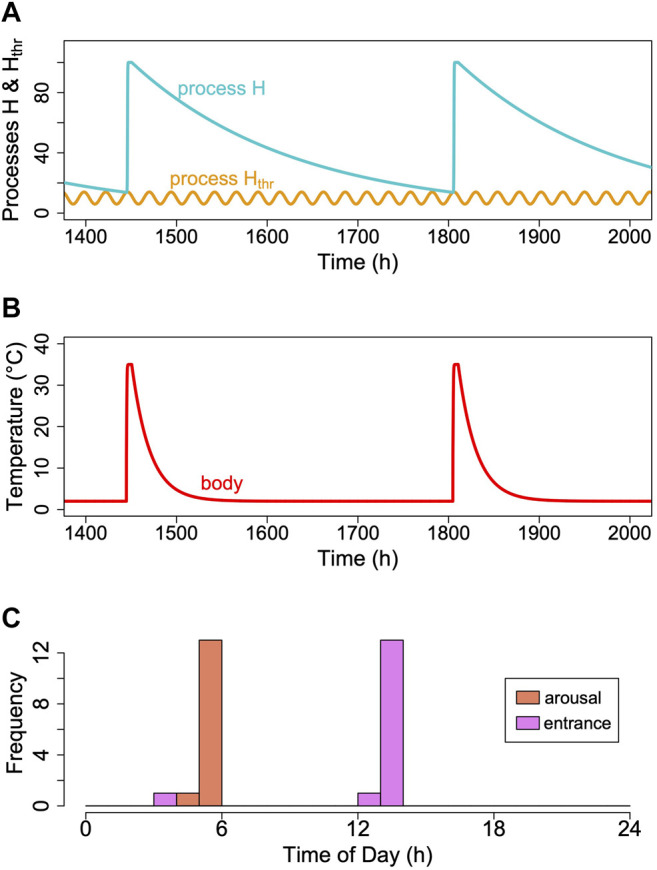
Introducing a circadian rhythmicity. A rhythm in the threshold process H_thr_ (orange line; **(A)**), leads to arousals starting in the rising phase of the circadian rhythm only. **(B)** Corresponding pattern of simulated T_b_. **(C)** A circadian rhythm in process H_thr_ leads to clustering of arousal and torpor entrance at certain circadian phases. This entrainment of torpor bout is not perfect, however, because even at a constant process H (in the example k = 0.0056), it may reach different cycles of H_thr_ over time, depending on their phase relationship (see also Supplementary Figure S2).

To our knowledge, there is only a single report on circadian rhythms of T_b_ that persist in deep torpor in ground squirrels (Grahn et al., 1994). Unfortunately, these results were never confirmed by a subsequent investigation in this species or another hibernator. However, as mentioned before, there is a considerable body of evidence pointing to an involvement of a circadian component in the control of torpor-arousal cycles (reviews in Körtner and Geiser (2000); Oklejewicz et al. (2001); Hut et al. (2002)). In several species, entrance into and/or arousal from torpor occur predominantly at certain phases of the circadian rhythm, especially when animals are kept under an LD cycle (Oklejewicz et al., 2001). Therefore, we modified the model by adding a circadian periodicity to process H_thr_ (Figure 10). The periods of cycles of process H_thr_ were kept constant and independent of T_b_, assuming a temperature-compensated circadian clock. Introducing this circadian periodicity immediately causes process H to reach process H_thr_ only during the rising phase of the threshold. This leads to arousals (and subsequent torpor entrances) to be concentrated around certain phases of the circadian rhythm (Figure 10C). Otherwise, adding a circadian rhythm to process H_thr_ did nothing to affect the outcome of the model.

## The Evolution and Adaptive Value of a Two-Process Mechanism

If the torpor-arousal cycle is dominated by an hourglass mechanism, this requires a major transition from a strictly circadian control of T_b_ and MR during the summer-active season to a hibernation-specific clock mechanism with a multi-day period that can even reach cycles lasting several weeks. This transition probably mirrors a major transition in the evolution of heterothermy in mammals. There seems to be a growing consensus that daily torpor, that is, shallow bouts of heterothermy that typically last several hours only (Ruf and Geiser, 2015), represents the more ancient trait, whereas prolonged hibernation is viewed as an advanced, secondary adaptation (Malan et al., 1996; Grigg et al., 2004; Lovegrove, 2012; Ruf and Geiser, 2015). Both euthermia and daily torpor are clearly governed by the circadian system, and bouts of daily torpor free-run with a circadian period under constant illumination (Lynch et al., 1980; Kirsch et al., 1991; Perret and Aujard, 2001). The multiday torpor of hibernators therefore requires either a complete loss of circadian rhythmicity or a decoupling of MR and T_b_ from the circadian pacemaker. Indeed, a circadian clock that persisted with daily “wake-up” signals during multi-day torpor would be maladaptive leading to increased metabolic costs, and evidence from various mammals points to the arrest of the central circadian clock during hibernation (Hut et al., 2002; Revel et al., 2007; Williams et al., 2012; Ikeno et al., 2017). If the circadian control of T_b_ and other physiological traits is temporarily - during hibernation - replaced by an hourglass clock, this raises the question of possible adaptive values of such a mechanism.

We can see several advantages of a clock mechanism that is directly coupled to MR, especially under conditions, such as underground hibernacula, in which entrainment with the natural light/dark cycle is unimportant. First, a dependence of TBD on MR allows the easy adjustment of the degree of energy saving, which is proportional to TBD, to environmental conditions. Not surprisingly then, TBD, while it is independent of body mass (Heldmaier et al., 2004; Ruf and Geiser, 2015; Ruf et al., 2021), is far from being randomly distributed. Ruf and Geiser (2015) found that both maximum and mean TBD among hibernating mammals significantly increase with the distance of the species’ distribution range center from the equator. As to be expected from the relationship between TMR and TBD (Figure 2) minimum TMR is also correlated with geographical latitude (r = −0.37, *p* < 0.01, n = 50, data from Table 1 in Ruf and Geiser (2015)). Since latitude of the distribution range can be seen as proxy of seasonality and harshness of the environment, this indicates that severe habitats select for lower TMR, which, given the hourglass mechanism, saves energy not just by itself but even more so by increasing TBD. Besides, it should be noted that mammals living at lower latitudes apparently avoid extremely long and deep torpor bouts. Whereas torpor results in enormous energy savings, it also apparently involves risks and trade-offs (Humphries et al., 2003; Munro et al., 2005; Bieber and Ruf, 2009; Bieber et al., 2014; Zervanos et al., 2014; Nowack et al., 2019).

Second, an MR-dependent hourglass allows for rapid adjustments of TBD to fluctuating environmental conditions, such as fluctuations in T_a_ (Figure 4). This applies not only to torpor phases, but also to arousals. For example, in the edible dormouse, which hibernates in relatively shallow burrows, decreases in burrow temperature lead to a profound shortening of the duration of arousals (Bieber and Ruf, 2009). This suggests that even within the same individuals MR affects not only the build-up of a metabolic imbalance, but also its clearance rate during IBE. Obviously, such a coupling would be highly adaptive because it reduces the time spent at euthermic T_b_ precisely when large T_b_-T_a_ gradients would make IBEs energetically most costly.

Third, an hourglass mechanism will allow hibernators to adjust both TBD and IBE duration to their own body condition and energy reserves. Many hibernators require sufficient body fat stores to survive winter (Arnold, 1993; Giroud et al., 2014). However, there is accumulating evidence that large surplus external or body energy reserves can cause hibernators to spend more time at euthermia, to shorten TBD, and to maintain higher minimum T_b_ in torpor (Humphries et al., 2003; Bieber et al., 2014; Zervanos et al., 2014). This latter observation may indicate that hibernators in good condition might shorten TBD by simply increasing minimal T_b_ in torpor (and hence speeding up MR and process H), while at the same time avoiding possible risks and adverse effects of extremely low T_b_ (cf., Giroud et al., 2013; Arnold et al., 2015; Nowack et al., 2019).

Thus, a flexible and adjustable hourglass mechanism may have several selective advantages. Also, this mechanism seems a more parsimonious explanation for characteristics of torpor-arousal cycles than postulating the existence of a non-temperature-compensated circadian clock outside the circadian masterclock in the nucleus suprachiasmaticus (SCN) (Malan, 2010). A plain hourglass mechanism would be sufficient to explain typical hibernation patterns (Figure 3), without any involvement of circadian rhythms. However, the two-process model proposed here also can explain how hibernators may retain some degree of circadian rhythmicity in the timing of arousals. This, however, should be adaptive only for species that are repeatedly exposed to natural lighting.

## Conclusion

In summary, the two-process model of torpor-arousal cycles is compatible with a number of phenomena observed in hibernating mammals, namely the inverse relationship between TMR and TBD, the dependency of TBD on T_a_ and T_b_, the modulation of torpor depth and duration within the hibernation season, and the absence of circadian rhythms in T_b_ or MR during torpor. The model does not require, but allows for, a circadian component that tends to synchronize arousals, which may be adaptive for certain hibernators that regularly return to above ground activity during winter. Future studies may prove the model insufficient or overly simplistic. However, we would argue that, given the significant relationship between the minimum rate of MR in torpor and TBD, there can be little doubt that torpor-arousal cycles are principally governed by the accumulation of a metabolic imbalance. Therefore, the logical, most important task ahead will be identification of the nature of process H. This may well prove to be difficult, because torpor could be associated with the depletion a multitude of metabolites, most of which will not be rate-limiting. However, one possible avenue for this search could be testing if some of the individual variation in TBD has a genetic basis, in addition to the effects of phenotypic variation. It is at least promising that the only hibernation trait investigated in this context, emergence date, in fact has substantial heritability (Lane et al., 2011). If this was also the case for TBD, it would allow screening for genes and metabolites that differ between genotypes that display different TBDs under identical environmental conditions.

## Data Availability

The original contributions presented in the study are included in the article/Supplementary Material, further inquiries can be directed to the corresponding author.
