# Supplementary material for: Hypothesis and Theory: A Two-Process Model of Torpor-Arousal Regulation in Hibernators
Source: Front Physiol. 2022 Jun 20;13:901270. doi: 10.3389/fphys.2022.901270 (PMC9266152; doi:10.3389/fphys.2022.901270)
Supplement: Supplementary file 1 [file DataSheet1.docx]

Supplemental Information

Hypothesis and theory: a two-process model of torpor-arousal regulation in hibernators

Thomas Ruf^1,2*^, Sylvain Giroud^1^, and Fritz Geiser^2^

^1^ *Department of Interdisciplinary Life Sciences, Research Institute of Wildlife Ecology, University of Veterinary Medicine, Vienna, Austria, Savoyenstr. 1, 1160 Vienna, Austria*

^2^ *Centre for Behavioural and Physiological Ecology, Zoology,
University of New England, Armidale, NSW 2531, Australia.*

**

Fig. S1. Torpor bout duration as a function of torpor metabolic rate in the Garden dormouse (*Eliomys quercinus).* Data from Ruf et al. (2021).

Fig. S2. (A) Predicted relationship between the period τ of rhythms in process H_thr and arousal-to-arousal (Ar-Ar) intervals. Intervals were computed by varying τ from 19 to 27 h. (B) When τ=23.9 h, process H (turquoise line) reaches process H_thr (orange line) during its 80th cycle in the model run shown. (C) If τ is further increased to 24.0 h, arousal is induced already by the 79th cycle of process H_thr. This behavior of the model leads to a sudden shortening of the arousal-to-arousal intervals and to the sawtooth pattern in (A)). In the simulation shown here, the maximum change in Ar-Ar intervals was only 6% of the mean. When analyzing real data from animals, which certainly will contain additional noise, it would be virtually impossible to detect such a subtle pattern. Not surprisingly then, Oklejewicz et al. (2001), comparing three genotypes of hamsters with τs of ~20, ~22, and ~24 h, respectively, found no differences in Ar-Ar intervals during hibernation.

Fig. S3. Histogram of TBDs determined in garden dormice kept under a 24 light-dark cycle (A). Real data were digitized from Fig. S2B in Daan (1973). (B) Simulated TBD from a model using a circadian rhythm in process T (τ=24.0 h, amplitude=4), and time constants k of process H randomly varying between 0.003 and 0.3. To create a model output that resembles real data, normally distributed noise (SD=2) was added to the simulated TBDs.

The model also predicts that, if these histograms were plotted for Ar-Ar intervals, rather than TBDs, the peaks would occur exactly at multiples of 24 hours. This is indeed the case in actual records from animals, for an example see the histogram of Ar-Ar periods in pocket mice (French, 1977). The decreasing amplitude of peaks with increasing TBD in Fig. S3 does not mean that the synchronizing effect of the circadian rhythm in process H_thr is diminished at long TBDs. This pattern merely occurs because, due to the exponential nature of process H, very long TBDs become exceedingly rare, even if the time constant k is uniformly distributed within a certain range. Another prediction of our model is that the discrete peaks in frequency distributions of TBDs from pooled data should vanish as soon as the circadian period of T is no longer identical in all animals. This is exactly what has been observed in distributions of TBD from animals kept under un-entrained conditions (c.f. Fig. 10A in Daan, 1973).

**R script of the model main function.**

Below is the main function of the model. To create different conditions (as in the figures) function ‘cycle’ is called with slightly different time constants (k) and thresholds (T). In the example the threshold has a circadian rhythmicity.

#"cycle" is the main function" simulating the alternation between torpor and IBE

cycle=function(H_thr,time,k=-0.005,kt=-0.02,kup=-8,ktup=-2,Tbmin=2,plot=TRUE){

H_thr=T

#T is the circadian threshold, call with default values

stp=median(diff(time))

H=rep(100,length(time)) # process H initially 100%

Tb=rep(35,length(time)) # initial Tb

ar=rep(0,length(time)) # arousal counter

kH=k

if(length(k)==1) kH=rep(k,length(time))

i=1

mx=length(time)

while (i<mx){

# enter torpor:

th=0

H0=100

Tb0=Tb[i]

repeat {

k=kH[i]

i=i+1

th=th+stp

H[i]=H0*exp(k*th)

Tb[i]=(Tb0-Tbmin)*exp(kt*th)+Tbmin

if (Tb[i]<30 & Tb[i-1]>=30) ar[i]= -1

if (H[i]<=T[i]) break # H reached T

if (i>=mx) break

}

# warm up:

th=0

H0=H[i]

Tb0=Tb[i]

repeat {

i=i+1

th=th+stp

H[i]= (100-H0) / (1 + exp(10 + kup*th))+H0

Tb[i]= (35-Tb0) / (1 + exp(1 + ktup*th))+Tb0

if (Tb[i]>30 & Tb[i-1]<=30) ar[i]=1

if (H[i]>=100) break # H returned to maximum

if (i>=mx) break

}

}

H=H[-i]

Tb=Tb[-i]

# If called with plot=T:

if (plot==TRUE){

n=min(length(time),length(T),length(H),length(Tb),length(ar))

time=time[1:n]

T=T[1:n]

H=H[1:n]

Tb=Tb[1:n]

ar=ar[1:n]

par(mar=c(5,5,1,2)) # set margins

par(mfrow=c(2,1)) # two plots

plot(time,T,ylim=c(0,100),type='l',col="blue",

ylab="T / H",xlab="Time (h)",cex.lab=1.5)

lines(time,H,type="l",col="red")

plot(time,Tb, type="l",

ylab="Tb (\u00b0C)",xlab="Time(h)",cex.lab=1.5)

}

# place all data in on frame and return

return(data.frame(time,T,H,Tb,ar))

}

#make a fictional time in the future from 15 Sep 2075 to 15 Apr 2076

tme=seq.POSIXt(ISOdate(2075,09,15), ISOdate(2076,4,15),by='6 min')

time=as.double(tme) #convert to double

time=time-time[1] #subtract first time

time=time/3600 #time in hours

quartz(width=12,height = 6) #make graphic window on Mac

# make rhythm

m=10 #mean level

A=2 #amplitude

phi=pi/2 #phasing

w=time/23.8*2*pi #rhythm is 23.8 h

T=m+A*cos(w-phi) # make rhythmic threshold

Results=cycle(T,time, k=-0.006, kt=-0.05,kup=-8, ktup=-4)

**R script to model TBD from TMR:**

#Example data digitized from Buck and Barnes (2000):

ta=c(-16,-8,-4,0,4,8,20)

mr=c(0.180,0.089,0.041,0.016,0.012,0.012,0.047)#original MRs

tbd=c(6.75,9.44,13.57,15.01,13.5,7.67,5.33)# data AGS

hh=1:4 #values <=0°C. Thermoregulation

t=1:1000

tbdur=function(k){ #make a function that determines H and the duration of TBD from k

H=100*exp(-k*t)

ix=min(which(H<10))

return(ix/24)

}

mreal=mr

err=NULL

count=0

p1=p2=p3=rep(0,10000)

for (j1 in seq(0.1,0.5,by=.02)){ #loop for the first factor.Used for high thermoregulatory values of MR.

if (length(hh)==0) j1=1 # If there is no thermoregulation.

TBD=NULL

mr[hh]=mreal[hh]*j1 #Multiply the hh values of measured MR with factor J1

print(mr)

for(j2 in seq(-0.005,0.005,by=0.0005)){

for(j3 in seq(0.1,1.2,by=0.005)){

TBD=NULL

for (m in mr){

k=j2+m*j3 #add j2 and multiply MR by j3

TBD=c(TBD,tbdur(k)) # calculate time in torpor

}

pred=TBD # predicted time in torpor

count=count+1

err=c(err,sum(abs(pred-tbd)^2)) # Error function

p1[count]=j1

p2[count]=j2

p3[count]=j3

}

}

}

opti=which.min(err) #find minimum error

p1[opti];p2[opti];p3[opti]

mr=mreal

mr[hh]=mreal[hh]*p1[opti]

kpred=p2[opti]+mr*p3[opti] # find optimal k for each MR

kpred

for (i in 1:length(mr)){

tbdpred[i]=tbdur(kpred[i]) #find predicted TBD

}

cbind(tbd,tbdpred) #compare actual, predicted

**R script containing data**

# Data Figs. 5 & 6

#Buck $ Barnes 2000

mr=c(0.180,0.089,0.041,0.016,0.012,0.012,0.047)

tbd=c(6.75,9.44,13.57,15.01,13.5,7.67,5.33)

hh=1:4

# Geiser & Kenagy 1988

mr=c(0.0466,0.0359,0.0262,0.1747)

tbd=c(4.66,8,10.7,8.4)

hh=4

# Geiser & Broome 1993

mr=c(0.0493,0.0384,0.272)

tbd=c(9.73,14,6.5)

hh=3

# Geiser et al. 1990

# E.amoenus

mr=c(0.043,0.025,0.03425)

tbd=c(4.9,8.67,4.7)

# S.saturatus

mr=c(0.03626,0.01653,0.02453)

tbd=c(7.12,11,8.77)

# Ruf et al. 2021

mr=c(0.0294,0.06157,0.1092) #means of long, middle, short bouts

tbd=c(10.70,8.04,3.69)

**R script to model seasonal change**

tme=seq.POSIXt(ISOdate(2075,09,1), ISOdate(2076,4,1),by='6 min')

time=as.double(tme)

time=time-time[1]

time=time/3600

m=10+90*exp(-0.005*time)

n=length(m)

first=m[1:(n/2)]

first=sort(first)

m[(n/2+1):n]=first

H_thr=m #please use min function cycle with these data

**R script to model circadian threshold**

tme=seq.POSIXt(ISOdate(2075,09,15), ISOdate(2076,4,15),by='6 min')

time=as.double(tme)

time=time-time[1]

time=time/3600

m=10

A=4

phi=pi/2

w=time/24*2*pi

H_thr=m+A*cos(w-phi)

result=cycle(H_thr,time, k=-0.0055, kt=-0.05,kup=-8, ktup=-4)

**R script to model effect of a small tau-shift**

tme=seq.POSIXt(ISOdate(2075,09,15), ISOdate(2076,4,15),by='6 min')

time=as.double(tme)

time=time-time[1]

time=time/3600

m=10

A=4

phi=pi/2

w=time/23.9*2*pi

H_thr=m+A*cos(w-phi)

res=cycle(H_thr,time, k=-0.0055, kt=-0.05,kup=-8, ktup=-4,plot=F)

quartz(width=6,height=7)

with (res, {

par(mar=c(5,5,1,2))

par(mfrow=c(2,1))

plot(time,C,ylim=c(0,30),type='l',col="orange2",ylab="Processes H & H_thr",xlab="Time (h)",xlim=c(1850,1910),cex.lab=1.3,lty=1,lwd=3)

lines(time,H,type="l",col="turquoise3",lwd=3)

text (1860,17,"process H",col="turquoise3",cex=1.3,srt=-4)

text(1883,4,"process H_thr",col="orange2",cex=1.3)

}

)

ix=which(max(C)-C<0.0001)

nc=1:length(ix)

text(time[ix],12,nc,cex=1.1)

text(1860,3,expression (paste(tau,' = 23.9 h')),cex=1.2)

phi=pi/2

w=time/24*2*pi

C=m+A*cos(w-phi)

res=cycle(C,time, k=-0.0055, kt=-0.05,kup=-8, ktup=-4,plot=F)

with (res, {

plot(time,C,ylim=c(0,30),type='l',col="orange2",ylab="Processes H & H_thr",xlab="Time (h)",xlim=c(1850,1910),cex.lab=1.3,lty=1,lwd=3)

lines(time,H,type="l",col="turquoise3",lwd=3)

text (1860,16,"process H",col="turquoise3",cex=1.3,srt=-4)

text(1890,4,"process H_thr",col="orange2",cex=1.3)

}

)

ix=which(C==max(C))

nc=1:length(ix)

text(time[ix],12,nc,cex=1.1)

text(1860,3,expression (paste(tau,' = 24.0 h')),cex=1.2)

**R script to simulate data from Daan (1973)**

cycle=function(CY,time,k=-0.005,kt=-0.02,kup=-8,ktup=-2,Tbmin=2,plot=T){

stp=median(diff(time))

H=rep(100,length(time))

Tb=rep(35,length(time))

ar=rep(0,length(time))

kH=k

if(length(k)==1) kH=rep(k,length(time))

i=1

mx=length(time)

while (i<mx){

#enter torpor:

th=0

H0=100

Tb0=Tb[i]

repeat {

k=kH[i]

i=i+1

th=th+stp

H[i]=H0*exp(k*th)

Tb[i]=(Tb0-Tbmin)*exp(kt*th)+Tbmin

if (Tb[i]<30 & Tb[i-1]>=30) ar[i]= -1

if (H[i]<=CY[i]) break # H reached CY

if (i>=mx) break

}

# warm up:

th=0

H0=H[i]

Tb0=Tb[i]

repeat {

i=i+1

th=th+stp

H[i]= (100-H0) / (1 + exp(10 + kup*th))+H0

Tb[i]= (35-Tb0) / (1 + exp(1 + ktup*th))+Tb0

if (Tb[i]>30 & Tb[i-1]<=30) ar[i]=1

if (H[i]>=100) break # H returned to maximum

if (i>=mx) break

}

}

H=H[-i]

Tb=Tb[-i]

n=min(length(time),length(CY),length(H),length(Tb),length(ar))

time=time[1:n]

CY=CY[1:n]

H=H[1:n]

Tb=Tb[1:n]

ar=ar[1:n]

if (plot==T){

par(mar=c(5,5,1,2))

par(mfrow=c(2,1))

plot(time,CY,ylim=c(0,100),type='l',col="blue",ylab="CY / H",xlab="Time (h)",cex.lab=1.5)

lines(time,H,type="l",col="red")

plot(time,Tb, type="l",ylab="Tb (\u00b0C)",xlab="Time(h)",cex.lab=1.5)

}

return(data.frame(time,H,Tb,ar))

}

tme=seq.POSIXt(ISOdate(2075,09,15), ISOdate(2076,4,15),by='6 min')

time=as.double(tme)

time=time-time[1]

time=time/3600

tme=as.POSIXlt(tme,tz="GMT")

dechour=tme$hour+tme$min/60

m=10

A=4

phi=pi/2

w=time/24*2*pi

H_thr=m+A*cos(w-phi)

set.seed(444)

time=time[1:10000]

TBD=ARAR=NULL

n=1000

rn=rexp(n,rate=0.1) # make exponential random distribution

rn=rn/max(rn)

rn=rn/2

rn=rn+0.008

for (i in 1:n) {

kerr=rn[i]

res=cycle(H_thr,time, k=-kerr, kt=-0.05,kup=-8, ktup=-4,plot=F)

up=res$time[which(res$ar==1)]

down=res$time[which(res$ar== -1)]

arar=median(diff(up))

tbd=median(up-down)

TBD=c(TBD,tbd)

ARAR=c(ARAR,arar)

}

par(mfrow=c(1,1))

aa=ARAR[ARAR<240]

aa=TBD[TBD<240]

library(MASS)

bb=aa+rnorm(length(aa),0,1.8)

bb=bb[bb<240]

bb=bb[bb>0]

quartz(width=7,height=6)

truehist(bb,breaks=seq(0,240,by=1), col='grey',xaxt='n',xlim=c(0,240),xlab='Torpor Bout Duration (h)', ylab="Proportion",cex.lab=1.5)

axis(side=1,at=seq(0,240,by=24))

box()

**Data availability**

Digitised data are available in an R-script (# 3) in this material.
